# Supplementary material for: Decomposition analysis of women’s empowerment-based inequalities in the use of maternal health care services in Ethiopia: Evidence from Demographic and Health Surveys
Source: PLoS One. 2023 Apr 27;18(4):e0285024. doi: 10.1371/journal.pone.0285024 (PMC10138853; doi:10.1371/journal.pone.0285024)
Supplement: S1 File — (DOCX) [file pone.0285024.s001.docx]

*************************************************************************************

S1 File. Stata lines of codes used in the article to prepare and use the design elements

* Datasets used: 2000, 2005, 2011 and 2016 EDHS

* Authors: Gebretsadik S et al

*Date: first written Jan 13, 2022; revised on March 28, 2023.

*************************************************************************************

// To give equal weight to each survey (before pooling the dataset):

scalar TOTWT=1000000 // any number is possible

quietly summarize v005

scalar T=r(sum)

gen weight_b=v005*TOTWT/T

// create new variables to be used later in creating unique clusters and strata (before pooling the data in each individual dataset):

gen survey=1 // for the first EDHS, 2000

gen survey=2 // for 2005 EDHS

gen survey=3 // for 2011 EDHS

gen survey=4 // for 2016 EDHS

// Duplicate this variable by name "year" for extra caution. Again, this is before pooling.

gen year="2000"

gen year="2005"

gen year="2011"

gen year="2016"

// confirm the right variable for strata: before pooling.

egen strata=group (v024 v025) // for 2016 EDHS. for the other EDHSs v023 is correct and we took it.

gen strata=v023 // for 2000-2011 EDHS

// generate unique strata and clusters:

egen strata_pooled=group(survey strata) //after pooling the dataset

egen cluster_pooled=group(survey v021) //after pooling the dataset

// svset

svyset cluster_pooled [pw= wt], strata (strata_pooled) singleunit(centered

svy: regression command

**********************************************************END.
