# Supplementary material for: Decomposition analysis of women’s empowerment-based inequalities in the use of maternal health care services in Ethiopia: Evidence from Demographic and Health Surveys
Source: PLoS One. 2023 Apr 27;18(4):e0285024. doi: 10.1371/journal.pone.0285024 (PMC10138853; doi:10.1371/journal.pone.0285024)
Supplement: S1 Checklist — (DOCX) [file pone.0285024.s002.docx]

STROBE Statement—checklist of items that should be included in reports of observational studies

|  | Item No. | Recommendation | Page  No. | Relevant text from manuscript |
| --- | --- | --- | --- | --- |
| **Title and abstract** | 1 | (*a*) Indicate the study’s design with a commonly used term in the title or the abstract | 1 | We indicated this by the word “survey” |
|  |  | (*b*) Provide in the abstract an informative and balanced summary of what was done and what was found | 2 &3 | The phrase “Ethiopia Demographic and Health Surveys” indicates that the design is survey (cross sectional study) |
| Introduction | | | |  |
| Background/rationale | 2 | Explain the scientific background and rationale for the investigation being reported | 4- 6 | The last section of introduction explains succinctly the knowledge gap this study tries to address: no comprehensive study on disparity in access to maternal health care using a validated women’s empowerment index as equity stratifier  -The study showed how improvement in women’s empowerment would be translated into equitable maternal health care services |
| Objectives | 3 | State specific objectives, including any prespecified hypotheses | 6 | The last paragraph of the introduction section |
| Methods | | | |  |
| Study design | 4 | Present key elements of study design early in the paper | 9-10 | -we have shown that the study is a national survey (cross sectional design).  -Complex sampling procedures were undertaken (samples were selected through stratified, two stage clustering sampling procedure).  -Enumerations areas were selected in first stage and households in second stage. |
| Setting | 5 | Describe the setting, locations, and relevant dates, including periods of recruitment, exposure, follow-up, and data collection | 7, 9, 10 | -settings and locations well narrated (see page 7 &8)  - Data collection-see page 9 &10  - periods of recruitment, exposure, follow-up-**does not apply as the study uses secondary survey data.** |
| Participants | 6 | (*a*) *Cohort study*—Give the eligibility criteria, and the sources and methods of selection of participants. Describe methods of follow-up  *Case-control study*—Give the eligibility criteria, and the sources and methods of case ascertainment and control selection. Give the rationale for the choice of cases and controls  ***Cross-sectional study***—Give the eligibility criteria, and the sources and methods of selection of participants | 9 &10 | -Women in the reproductive age group  - Once households are selected systematically in the second stage in the two-stage clustering sampling procedure, then all women in the reproductive age group were studied (**selection does not apply).** |
|  |  | (*b*) *Cohort study*—For matched studies, give matching criteria and number of exposed and unexposed  *Case-control study*—For matched studies, give matching criteria and the number of controls per case |  |  |
| Variables | 7 | Clearly define all outcomes, exposures, predictors, potential confounders, and effect modifiers. Give diagnostic criteria, if applicable | 10-13 | Dependent, confounding and exposure variables were defined to the sufficient detail. |
| Data sources/ measurement | 8* | For each variable of interest, give sources of data and details of methods of assessment (measurement). Describe comparability of assessment methods if there is more than one group | *9-13* | *-*all variables/data were collected from participants’ self-report *except* wealth which is created based on observable household features and possessions. |
| Bias | 9 | Describe any efforts to address potential sources of bias | 13- 17 | -The complex nature of the EDHS data was taken into account to adjust the unequal probability of selection, clustering and stratification. Otherwise, the findings would be biased.  In the pooled data, our model was controlled for “year” of the surveys to capture the effect of time on the services.  For the bounded variables like ANC, we used Erreygers index instead of the standard concentration index to avoid producing erroneous findings.  Missing and do not know replies were handled based on standard approaches. |
| Study size | 10 | Explain how the study size was arrived at | 10 | A trade-off must be made between the required survey precision and the available budget when determining the ideal sample size  The computation of the ideal sample size ultimately boils down to calculating the ICC. The optimal sample take is an increasing function of cost ratio and a decreasing function of ICC.  see the paragraph immediately above the “variables” section for the detail. |

Continued on next page

| Quantitative variables | 11 | Explain how quantitative variables were handled in the analyses. If applicable, describe which groupings were chosen and why | 13-14 | The choices on the grouping of the variables are made based on a number of considerations such as experience or field expertise (for example, it is known that utilization of maternal health care services varies by whether or not the receipt is adolescent mother), works of prior studies on this same area, and sample size.  We relied heavily on the conceptual, rather than statistical, understandings to determine whether the variables could potentially be confounders by using the three criteria of confounding. We avoided reliance on p-values to detect confounding variables as the sheer existence of statistically significant findings does not serve this objective |
| --- | --- | --- | --- | --- |
| Statistical methods | 12 | (*a*) Describe all statistical methods, including those used to control for confounding | 15-18 | -methods of analysis was explained in much detail  We used appropriate regression model to adjust for the effect of confounding. |
|  |  | (*b*) Describe any methods used to examine subgroups and interactions |  | Does not apply. We have no interaction in our models. |
|  |  | (*c*) Explain how missing data were addressed | 18 | We used the DHS guide to handle missing as well as do not know responses. |
|  |  | (*d*) *Cohort study*—If applicable, explain how loss to follow-up was addressed  *Case-control study*—If applicable, explain how matching of cases and controls was addressed  *Cross-sectional study*—If applicable, describe analytical methods taking account of sampling strategy | 17 | We described our analytical methods that fully accounts the sampling strategy |
|  |  | (*e*) Describe any sensitivity analyses |  | We did not do sensitivity analyses |
| Results | | | | |
| Participants | 13* | (a) Report numbers of individuals at each stage of study—eg numbers potentially eligible, examined for eligibility, confirmed eligible, included in the study, completing follow-up, and analysed |  | Our study design is cross sectional and we used secondary data. |
|  |  | (b) Give reasons for non-participation at each stage |  |  |
|  |  | (c) Consider use of a flow diagram |  |  |
| Descriptive data | 14* | (a) Give characteristics of study participants (eg demographic, clinical, social) and information on exposures and potential confounders |  | We provided this information in Table 1 |
|  |  | (b) Indicate number of participants with missing data for each variable of interest |  | We already handled missing based on DHS guide to statistics |
|  |  | (c) *Cohort study*—Summarise follow-up time (eg, average and total amount) |  |  |
| Outcome data | 15* | *Cohort study*—Report numbers of outcome events or summary measures over time |  |  |
|  |  | *Case-control study—*Report numbers in each exposure category, or summary measures of exposure |  |  |
|  |  | *Cross-sectional study—*Report numbers of outcome events or summary measures | *Throughout; but mainly in tables (at the end of the manuscript)* | We reported the summary measures in tables and in texts as well |
| Main results | 16 | (*a*) Give unadjusted estimates and, if applicable, confounder-adjusted estimates and their precision (eg, 95% confidence interval). Make clear which confounders were adjusted for and why they were included |  | We provided confounder-adjusted estimates in Tables mainly. We put the tables at the end of the manuscript |
|  |  | (*b*) Report category boundaries when continuous variables were categorized | See tables at the end | We described how age is grouped in to two categories in “variables” section. All other variables were categorical in the dataset already. |
|  |  | (*c*) If relevant, consider translating estimates of relative risk into absolute risk for a meaningful time period |  | Does not apply. |

Continued on next page

| Other analyses | 17 | Report other analyses done—eg analyses of subgroups and interactions, and sensitivity analyses |  | We have no such analyses |
| --- | --- | --- | --- | --- |
| Discussion | | | | |
| Key results | 18 | Summarise key results with reference to study objectives | 27 | We did this. See the opening paragraphs of the discussion |
| Limitations | 19 | Discuss limitations of the study, taking into account sources of potential bias or imprecision. Discuss both direction and magnitude of any potential bias | 32 | We did this. See the concluding paragraph of the discussion |
| Interpretation | 20 | Give a cautious overall interpretation of results considering objectives, limitations, multiplicity of analyses, results from similar studies, and other relevant evidence | 27-32 | We did this |
| Generalisability | 21 | Discuss the generalisability (external validity) of the study results | 33 | We did this. |
| Other information | |  | | |
| Funding | 22 | Give the source of funding and the role of the funders for the present study and, if applicable, for the original study on which the present article is based |  |  |

*Give information separately for cases and controls in case-control studies and, if applicable, for exposed and unexposed groups in cohort and cross-sectional studies.

**Note:** An Explanation and Elaboration article discusses each checklist item and gives methodological background and published examples of transparent reporting. The STROBE checklist is best used in conjunction with this article (freely available on the Web sites of PLoS Medicine at http://www.plosmedicine.org/, Annals of Internal Medicine at http://www.annals.org/, and Epidemiology at http://www.epidem.com/). Information on the STROBE Initiative is available at www.strobe-statement.org.
