# Supplementary material for: Decomposition analysis of women’s empowerment-based inequalities in the use of maternal health care services in Ethiopia: Evidence from Demographic and Health Surveys
Source: PLoS One. 2023 Apr 27;18(4):e0285024. doi: 10.1371/journal.pone.0285024 (PMC10138853; doi:10.1371/journal.pone.0285024)
Supplement: Attachment — Submitted filename: Response to Reviewers.docx [file pone.0285024.s003.docx]

Journal requirements:

When submitting your revision, we need you to address these additional requirements.

1. Please ensure that your manuscript meets PLOS ONE's style requirements, including those for file naming.

Response: Thank you. We revised and prepared the materials in accordance with PLOSE ONE’s style requirement.

2. We note that you have indicated that data from this study are available upon request. PLOS only allows data to be available upon request if there are legal or ethical restrictions on sharing data publicly.

In your revised cover letter, please address the following prompts:

a) If there are ethical or legal restrictions on sharing a de-identified data set, please explain them in detail (e.g., data contain potentially sensitive information, data are owned by a third-party organization, etc.) and who has imposed them (e.g., an ethics committee). Please also provide contact information for a data access committee, ethics committee, or other institutional body to which data requests may be sent.

Response: Thank you. We have clarified the ambiguity of the statement on the availability of the data we based our study on. We used the EDHS data which are made public by the DHS programme. There is no ethical or legal restriction to accessing the dataset. The “upon request” phrase has now been omitted.

b) If there are no restrictions, please upload the minimal anonymized data set necessary to replicate your study findings as either Supporting Information files or to a stable, public repository and provide us with the relevant URLs, DOIs, or accession numbers. For a list of acceptable repositories, please see <http://journals.plos.org/plosone/s/data-availability#loc-recommended-repositories>.

We will update your Data Availability statement on your behalf to reflect the information you provide.

Response: Thank you. As we have said above, the EDHS data are available to the public domain and access to it is unrestricted. There are no data we owned privately. We have shown this in our revised cover letter.

3. PLOS requires an ORCID iD for the corresponding author in Editorial Manager on papers submitted after December 6th, 2016. Please ensure that you have an ORCID iD and that it is validated in Editorial Manager. To do this, go to ‘Update my Information’ (in the upper left-hand corner of the main menu), and click on the Fetch/Validate link next to the ORCID field. This will take you to the ORCID site and allow you to create a new iD or authenticate a pre-existing iD in Editorial Manager. Please see the following video for instructions on linking an ORCID iD to your Editorial Manager account: <https://www.youtube.com/watch?v=_xcclfuvtxQ>

Response: Thank you. I updated my existing ORCID iD.

4. Please include your full ethics statement in the ‘Methods’ section of your manuscript file. In your statement, please include the full name of the IRB or ethics committee who approved or waived your study, as well as whether or not you obtained informed written or verbal consent. If consent was waived for your study, please include this information in your statement as well.

Response: Thank you. We have now added ethics statement in the method section.

5. Please review your reference list to ensure that it is complete and correct. If you have cited papers that have been retracted, please include the rationale for doing so in the manuscript text, or remove these references and replace them with relevant current references. Any changes to the reference list should be mentioned in the rebuttal letter that accompanies your revised manuscript. If you need to cite a retracted article, indicate the article’s retracted status in the References list and also include a citation and full reference for the retraction notice.

Response: Thank you. The references are correct.


Reviewers' comments:

Reviewer's Responses to Questions

**Comments to the Author**

1. Is the manuscript technically sound, and do the data support the conclusions?

The manuscript must describe a technically sound piece of scientific research with data that supports the conclusions. Experiments must have been conducted rigorously, with appropriate controls, replication, and sample sizes. The conclusions must be drawn appropriately based on the data presented.

Reviewer #1: Yes

Response: Thank you.

2. Has the statistical analysis been performed appropriately and rigorously?

Reviewer #1: Yes

Response: Thank you.

3. Have the authors made all data underlying the findings in their manuscript fully available?

The [PLOS Data policy](http://www.plosone.org/static/policies.action#sharing) requires authors to make all data underlying the findings described in their manuscript fully available without restriction, with rare exception (please refer to the Data Availability Statement in the manuscript PDF file). The data should be provided as part of the manuscript or its supporting information, or deposited to a public repository. For example, in addition to summary statistics, the data points behind means, medians and variance measures should be available. If there are restrictions on publicly sharing data—e.g. participant privacy or use of data from a third party—those must be specified.

Reviewer #1: Yes

Response: Thank you.

4. Is the manuscript presented in an intelligible fashion and written in standard English?

PLOS ONE does not copyedit accepted manuscripts, so the language in submitted articles must be clear, correct, and unambiguous. Any typographical or grammatical errors should be corrected at revision, so please note any specific errors here.

Reviewer #1: Yes

Response: Thank you.

5. Review Comments to the Author

Please use the space provided to explain your answers to the questions above. You may also include additional comments for the author, including concerns about dual publication, research ethics, or publication ethics. (Please upload your review as an attachment if it exceeds 20,000 characters)

Reviewer #1: The authors present an interesting retrospective analysis and use women's empowerment as an entry point to explore inequalities in maternal health services. The study is very well written and provides important data to readers of the journal PLOS ONE.

Response: Thank you.

A few suggestions are included below:
1) On page 13, According to the instructions, the article's core variable, women's empowerment, was formed based on SWPER GLOBAL. Still, its formation as an important variable needs to be clearly described. If PCA began it, please provide data for each load to illustrate the applicability of the empowerment scale in Ethiopia. If the calculation was done directly using the code supplied by SWPER GLOBAL, it should also be stated that no PCA was performed but that the composite index method was used for direct formation.

Response: Thank you for pointing this issue out. What we wanted to portray was that the authors of the SWPER Global themselves used PCA to drive scores of each variable. By PCA, we only mean that they used it as part of their formation of the composite indices. We simply utilized the codes supplied by them to create these indices in our dataset. We have now clarified this point in the revised paper.

2) On page 30，Education is used as an explanatory variable and a dimension constituting social independence. Education’s prediction of the social independence component may not be scientifically valid, by which I mean, is there literature to support you in doing so?

Response: We want to thank you again for your comment. Education is not a predictor of social independence but maternal health services. We took into account in our model maternal education because we believed that it could potentially muddle the empowerment-maternal service usage connection. The rationale is that education is one of the many elements that make up the social independence domain. The SWPER Global authors recognize that education can affect women’s empowerment and that is why education has become one of the constituting components of women empowerment, mainly the social independence dimension. A woman has a greater likelihood of achieving social empowerment the more educated she is. So, education and social independence have a link between each other.

Social independence refers to women's empowerment or capacity to achieve their objectives. We also made an effort to clarify what the other domains pertain to so that readers could readily comprehend them, using your insightful suggestion about the need to elaborate more on social independence. Therefore, the nexus between maternal education and women empowerment (social independence) is just association, and in this study, this association was known by looking at the concentration index of education with respect to the ranking variable of social independence. A +ve concentration index of education (especially the higher educational status) means that more of the higher education is concentrated among women who are more empowered.

3) In Table 1, one of the brackets for the Attitude towards violence variable is incorrectly used.

Response: Thank you. We have now corrected it.
